# Cross-linked hyaluronan gel inhibits the growth and metastasis of ovarian carcinoma

**DOI:** 10.1186/s13048-018-0394-z

**Published:** 2018-03-06

**Authors:** Ji Pang, Pengcheng Jiang, Ying Wang, Lu Jiang, Hai Qian, Yan Tao, Ruxia Shi, Jizong Gao, Yongchang Chen, Yan Wu

**Affiliations:** 1 Department of Physiology, School of Medicine, Jiangsu University, Zhenjiang City, Jiangsu Province 212013 People’s Republic of China; 2 Department of Obstetrics and Gynecology, Changzhou Second People’s Hospital Affiliated to Nanjing Medical University, Changzhou City, Jiangsu Province China; 3 R & D Department, Changzhou BioRegen Biomedical (Changzhou) Co., Ltd., Changzhou City, Jiangsu Province China

**Keywords:** HyaRegen gel, Ovarian carcinoma, Migration, Invasion, Growth

## Abstract

**Background:**

The recurrence, metastasis and poor prognosis are important characteristics of ovarian carcinoma (OC), which are associated with exfoliation of cells from the primary tumor and colonization of the cells in pelvic cavity. On the other hand, the life quality of the patients undergoing surgical resection of OC was influenced by postoperative adhesions. Therefore, preventing postoperative implant tumor and adhesion may be effective methods to improve OC treatment. HyaRegen Gel, a cross-linked hyaluronan gel (CHAG), has been widely used as an anti-adhesive agent following pelvic operation in clinic. However, whether it can affect the implantation and growth of OC cells or not is still not clear.

**Methods:**

Migration and invasion assays were applied to detect the effect of CHAG on migration and invasion of OC cells. Western blotting was performed to detect the phosphorylation/activation of EGFR and ERK, and the expression of PCNA and MMP7. Pull down assay was used to analyze the effect of CHAG on the activation of small G protein Rac1. Nude mice implantation tumor model was applied to observe the effect of CHAG on implantation tumor of OC cells.

**Results:**

The results of in vitro experiments showed that CHAG suppressed both basic and EGF-induced migration and invasion of OC cells, blocked the activation of EGF-initiated EGFR activation, inhibited downstream signal transduction of EGFR, and decreased expression of proliferation and migration/invasion related proteins. Meanwhile, results of in vivo experiments showed that CHAG not only inhibited the formation of implantation tumor of OC cells but also delayed the of the growth of the tumors.

**Conclusions:**

CHAG inhibited migration, invasion and proliferation of OC cells in vitro, and suppressed development of implantation tumor of OC in vivo. This made it as both anti-tumor and anti-adhesion agents.

**Electronic supplementary material:**

The online version of this article (10.1186/s13048-018-0394-z) contains supplementary material, which is available to authorized users.

## Background

Ovarian cancer (OC) represents the most lethal malignancy of female reproductive system and the poor prognosis of OC is often attributable to late diagnosis, postoperative metastasis and recurrence [1, 2]. Many factors affect the prognosis, for example, post-operative adhesion [3, 4], malignant cells dropping off from the primary during surgical resection, and then implanting in abdominopelvic cavity. Therefore, taking measures to prevent **abscission  of tumour cells** and choosing suitable anti-adhesion produces to reduce postoperative adhesions are equally important for favorable prognosis [5–7].

Hyaluronic Acid (HA) is made up of glucuronic acid and N-acetylglucosamine disaccharide units. It is abundant in the skin and connective tissues, with a turnover time from several hours to a few days according to tissues [8]. HA plays crucial roles in cell motility, cell adhesion, organization of tissue architecture, and cell proliferation processes [9, 10]. Owing to its biological properties, HA has several clinical applications in aesthetic surgery, dentistry, dermatology, ophthalmology and orthopedics [8]. **Clinical** research showed that injection of hydrogel containing HA and chitosan could serve as an ideal barrier to prevent postoperative tissue adhesion [11]. **A** randomly-controlled trial reported that a new crosslinked gel (CHAG) was efficient and safe in reducing adhesions after gynecologic laparoscopic surgeries in clinical practice [12]. Additionally, absorbable auto-crosslinked hyaluronan gel could prevent intrauterine adhesion (IUA), decrease adhesion severity, and improve menopause postoperatively, indicating that this absorbable gel could be proposed as a barrier for preventing IUA after intrauterine procedures [13]. In a word, hyaluronan might be applied in resection of pelvic tumor for anti-adhesion, but it was still not clear whether the gel might be a potential stimulus for tumor metastasis and growth or not? In other words, is it safe for HA gel to be applied in tylectomy of pelvic tumor? Our previous study showed that CHAG suppressed colonization, growth and metastasis of gastric cancer cells [14]. However, it is still worthy to investigate whether CHAG has the similar effect on female pelvic tumor, such as OC, and whether CHAG is safe enough to be applied in OC operation for preventing post-operatic adhesion. Therefore, the present work was designed to address the above question. Our results showed that CHAG suppressed the migration and invasion of ovarian cancer cells, and delayed the development OC implantation tumor through blocking EGF-stimulated activation of EGFR and its downstream signal transduction.

## Methods

### Cell lines and mice

Human ovarian cancer cell line A2780 was purchased from CHI Scientific Inc. (Maynard, MA, USA) and human ovarian cancer cell line SKOV3 was purchased from Institute of Cell Biology (Shanghai, China). The cells were cultured in Dulbecco’s modified Eagle’s medium (DMEM), supplemented with 10% heat-inactivated fetal bovine serum (FBS; Gibco; Thermo Fisher Scientific, Inc., Waltham, MA, USA), 100 U/ml penicillin and 100 μg/ml streptomycin (HyClone; GE Healthcare Life Sciences, Logan, UT, USA), in a humidified incubator at 37 °C and 5% CO_2_. Female nude BALB/c mice (with the age of 6 weeks) were purchased from the Animal Center of Yangzhou University (Yangzhou, Jiangsu Province, China) and maintained in the Animal Center of Jiangsu University in compliance with the Guide for the Care and Use of Laboratory Animals (NIH,76 FR 91; May 11, 2011).

### Reagents

CHAG was provided by BioRegen Biomedical (Changzhou) Co., Ltd. (Changzhou, Jiangsu Province, China). Antibodies against β-actin (cat. no. sc-4778; dilution, 1:1000), Rac 1 (cat. no. sc-24,567; dilution, 1:500), and matrix metalloproteinases (MMP) 7 (cat. no. sc-80,205; dilution, 1:500) were purchased from Santa Cruz Biotechnology, Inc. (Dallas, TX, USA). The antibodies against proliferating cell nuclear antigen (PCNA; cat. no. #13110; dilution, 1:1000), phosphorylation (p)-EGFR (Tyr1068) (cat. no. #3777; dilution, 1:1000), p-EGFR (Tyr1173) (cat. no. #4407; dilution, 1:1000), p-Akt (Ser473) (cat. no. #4060; dilution, 1:1000) and p-Erk1/2 (Thr202/Tyr204) (cat. no. #4370; dilution, 1:1000) were purchased from Cell Signaling Technology, Inc. (Danvers, MA, USA). Horseradish peroxidase (HRP)-conjugated goat anti-rabbit and goat anti-mouse secondary antibodies (cat. nos. 111–035-003 and 115–035-003; dilution, 1:10,000) were purchased from Jackson Immuno Research Laboratories, Inc. (West Grove, PA, USA). Transwell plates were purchased from Corning Incorporated (Corning, NY, USA). ECM Gel was purchased from Sigma-Aldrich, Inc. (St. Louis, MO, USA). Recombinant Human EGF (rEGF) was purchased from PeproTech, Inc. (Rocky Hill, NJ, USA). Enhanced chemiluminescence (ECL) reagents were from EMD Millipore (Billerica, MA, USA).

### Cell migration assay

Cells in logarithmic growth phase were cultured in FBS-free DMEM for 12 h. After trypsin digestion and centrifugation, 5 × 10^5^/mL cells were re-suspended in different concentrations of CHAG in FBS-free DMEM medium. EGF (100 ng/mL) was added to stimulate the migration of the cells.** Three hundred  μL** of the above cell suspension was added to the upper chamber of Transwell plate and 500 μL of DMEM containing 10% FBS was added to the lower chamber. The migration time for the cells was 12 h. At the end of the migration, the cells retained on the upper surface the membrane were swapped off and the cells migrated onto the lower surface of the membrane were stained with Giemsa and then counted under inverted microscopy.

### Cell invasion assay

Cell invasion assays were same as described in cell migration assay except that the membrane of the upper chamber was coated with 60 μL of 1.125 μg/μL ECM Gel before adding cell suspension and the invasion time for the cells was 24 h.

### The model of transplantation tumor of ovarian cancer cells in nude mice and treatment

Specific Pathogen Free (SPF) grade female BALB/c nude mice with weights of 8.76 ± 1.34 g were maintained in a SPF barrier system. In order to increase the rate of tumor formation, 1 × 10^7^ cells suspended in 400 μL of PBS were implanted into one mouse by subcutaneous injection. After 2 weeks, the tumors were collected and ground into cell suspension with glass homogenizer. The dispersed cells were continually cultured to an adequate number. After trypsin digestion, 1 × 10^7^ cells suspended in 400 μL of PBS were implanted into each mouse by intra-peritoneal cavity injection. After 2 h, 400 μL PBS or 400 μL PBS containing **20 μg CHAG** were injected into the peritoneal cavity and the injection was repeated once every week until the 4th weeks. At the end of the experiment, the animals were euthanized, and the tumors were collected and weighed.

### Western blotting

The A2780 and SKOV3 cells were accordingly treated and the whole cell lysates were harvested. All procedures of Western blotting were performed following the manufacturer’s protocol (Bio-Rad, Hercules, CA). The primary antibodies were incubated over night at 4 °C, and the corresponding secondary antibodies were incubated for 1 h at room temperature. Protein bands were showed by ECL reagents.

### “Pull-down” analysis of active small G protein Rac1

The activity of Rac1 was detected by Pull-down method. Cells (3 × 10^6^/mL) were cultured in 10 cm dishes with serum-free DMEM overnight. Then, the cells were treated with 500 μg/mL or 1000 μg/mL CHAG for 1 h and then stimulated with 100 ng/ml EGF for 5 min. Finally, the cells were harvested with lysis buffer. After centrifugation at 12000 g, 4 °C for 10 min, 20 μL of supernatant was used as the control of the loading. The remaining supernatants were incubated with 100 μL of glutathione glucan beads with GST-Pak1 protein binding domain (GST-PBD) at 4 °C for 1 h. Finally, the activated Rac1 bound to the beads and total Rac1 in cell extracts was detected by Western blotting.

### Statistical analysis

All experiments were performed in triplicate. Data are expressed as means ± standard deviation (SD). Statistical analysis was performed using a two-tailed ANOVA with SPSS statistical software. Student’s *t* test was performed if equal variance was ascertained in two groups by *F* test. *P* < 0.05 was considered significant.

## Results

### CHAG inhibits basic and EGF-induced migration and invasion of ovarian cancer cells

**Fig. 1:**
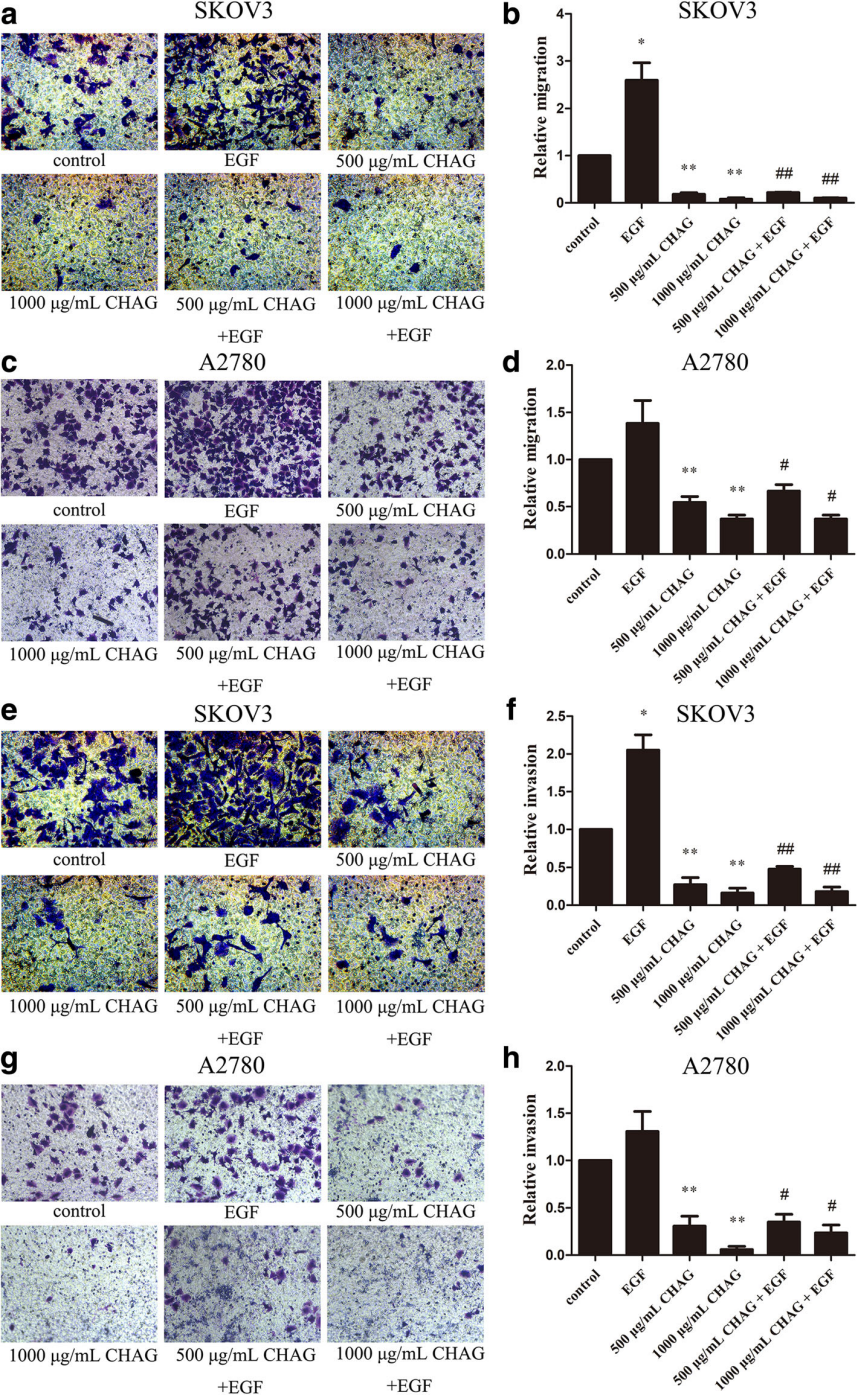
CHAG inhibits EGF-induced migration and invasion activities of ovarian cancer cells. **a**-**d** Migration activity of A2780 and SKOV3 cells. **e**-**h** Invasion activity of A2780 and SKOV3 cells. **a**, **c**, **e** and **g** were representative photomicrographs of cells stained by Giemsa (200×). **b**, **d**, **f** and **h** represent the folds of cells’ migration or invasion in the corresponding groups. Data are showed as means ± SD from 3 independent experiments. (*P < 0.05, **P < 0.01, compared with control group; ^##^*P* < 0.01, compared with EGF group)

The results of Transwell migration assay showed that compared to the control group, both 500 and 1000 μg/mL CHAG significantly inhibited the migration of A2780 and SKOV3 cells without stimulation of growth factors (Fig. 1a-d, *P*< 0.05). Furthermore, CHAG also inhibited the EGF-induced migration, and especially, 1000 μg/mL CHAG had a significant inhibitory effect (Fig. 1a-d, *P*< 0.05). Similar results were observed in invasion assay (Fig. 1e-h, *P*< 0.05). The above results indicated that CHAG had an inhibitory function on migration and invasion activities of ovarian cancer cells.

### CHAG inhibits the growth of implantation tumor originated from ovarian cancer cells

**Fig. 2:**
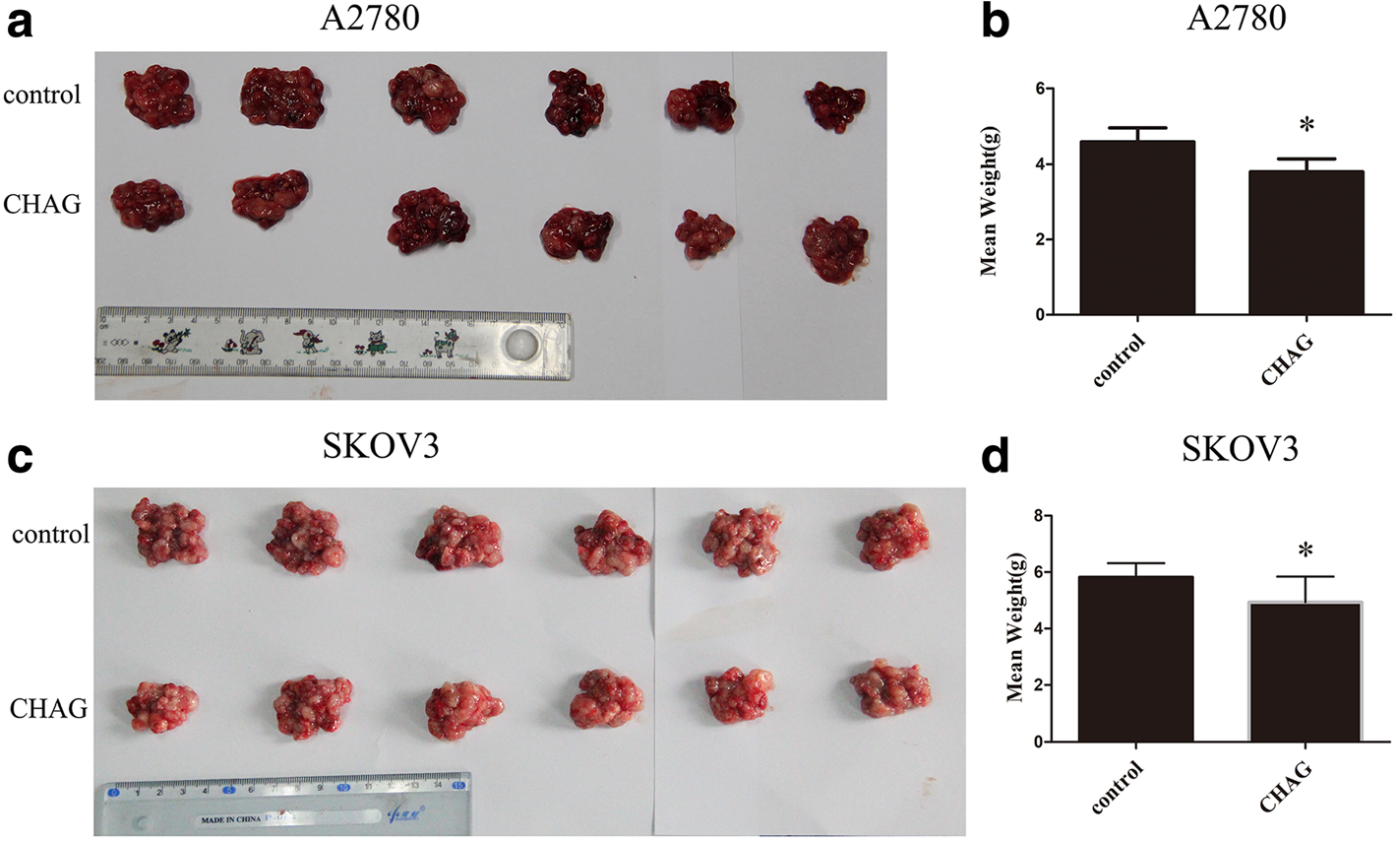
CHAG inhibits growth of ovarian cancer cell in pelvic cavity. **a** and **c** The tumors from the control and CHAG groups were shown. **b** and **d** The weight of the tumors in corresponding group. The data were shown as means ± SD. (*P < 0.05, compared with control group)

To investigate the effect of CHAG on the in vivo growth of cancer cells, ovarian cancer cells were implanted into the pelvic cavity of nude mouse. CHAG was administrated into pelvic cavity 2 h later, and then the administration was repeated once per week for 4 weeks. At the end of the experiment, the mice were executed and the weights of the transplantation tumors were measured. The results showed that CHAG treatment significantly decreased the weight of transplantation tumor of A2780 and SKOV3 cells (Fig. 2), demonstrating that CHAG had an inhibitory effect on the growth of transplanted ovarian cancer cells.

### CHAG inhibits the activation of EGFR and the EGF/EGFR-initiated signalings in ovarian cancer cells

**Fig. 3:**
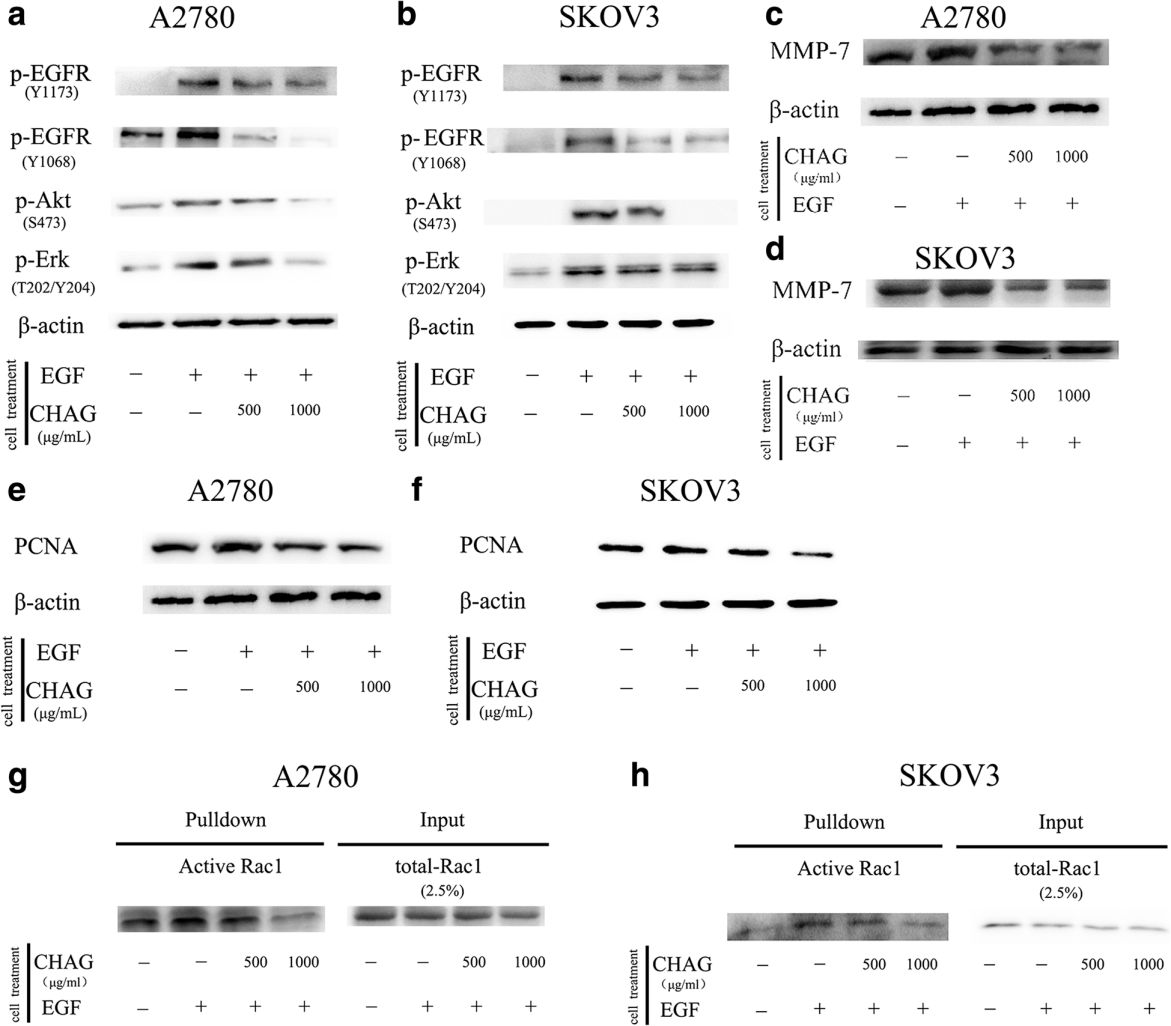
CHAG blocks activation of EGFR and its downstream signaling molecules, and inhibits EGF-induced expression of MMPs and PCNA in OC cells. **a** and **b** CHAG inhibited phosphorylation/activation of EGFR, Akt and ERK in A2780 and SKOV3 cells. The cells were cultured in serum free DMEM overnight and treated with 500 or 1000 μg/mL CHAG solutions for 1 h and then treated with 100 ng/ml EGF for 5 min. The cellular lysates were subjected to Western blotting. **c** and **d** Detection of the expression of MMP7 in A2780 and SKOV3 cells by Western blotting. In EGF group, the cells were treated with 100 ng/ml EGF for 24 h. In the CHAG + EGF groups, the cells were treated with 500 or 1000 μg/mL CHAG and 100 ng/mL EGF for 24 h. The cells were harvested and the lysates were subjected to Western blotting with anti-MMP7 antibodies. **e** and **f** Western blotting detection of the expression of PCNA in A2780 and SKOV3 cells. The cells were treated same as described in panel **c** and **d**. **g** and **h** CHAG blocked the activation of Rac1. The pull-down results were representatives of three independent experiments

Epidermal growth factor receptor (EGFR) is a key signaling molecule that drives cellular proliferation, migration, and invasion [15]. Some reports showed that high EGFR expression found in ovarian tumors [16] and EGFR signaling was involved in promoting ovarian cancer cell proliferation [17]. However, there is still some conflicting reports. For example, some data favored EGFR as a reliable marker of survival or responsiveness to therapy [18, 19] but others did not [20, 21], and some reports indicated that using EGFR inhibitors in ovarian cancer patient had not shown favorable clinical outcomes [15, 22, 23]. Thus, in this paper,** it was investigated** whether CHAG inhibited the development of ovarian cancer cells via abrogation of activation of EGFR and EGF/EGFR mediated signaling cascades. The result demonstrated both A2780 and SKOV3 expressed EGFR (Additional file 1: Figure S1), and EGF treatment (100 ng/mL, 5 min) led to an increase of Tyr1068 and Tyr1173 phosphorylation of EGFR, and pre-treatment with CHAG (500 and 1000 μg/mL) efficiently inhibited the EGF-induced phosphorylation of EGFR (Fig. 3a, b), indicating that CHAG inhibited EGF-induced activation of EGFR. Additionally, Western blotting results showed that EGF treatment (100 ng/mL, 5 min) caused significant increase of phosphorylation/activation of Akt and ERK, which were main signaling components downstream of EGFR. Treatment with CHAG blocked EGF-induced phosphorylation/activation of Akt and ERK (Fig. 3a, b). These results confirmed that CHAG could inhibit OC cells through blocking activation of EGFR and its downstream signalings.

### CHAG inhibits the activation of small G protein Rac1

Small G protein Rac1 is the **chief** member of Rho family which play important role in regulating migration of cancer cells [24]. Some of EGFR-mediated signal transduction can activate them and thereafter stimulate cell migration [25–27]. Therefore, it is worthy to investigate the inhibitory effect of CHAG on activation of Rac1. In this study, the “Pull-down” assay was performed to detect the level of active (GTP-bound) Rac1. The results showed that treatment with EGF (100 ng/mL, 5 min) increased the amount of GTP-bound/active Rac1. Pre-treatment with CHAG efficiently restrained the stimulating effects of EGF on the activation of the small G protein in both A2780 and SKVO3 (Fig. 3g, h).

### CHAG inhibits the expression of metastasis and proliferation related proteins.

To get more evidence for the inhibition of CHAG on the migration, invasion and proliferation of OC cells, the proliferation marker, proliferating cell nuclear antigen (PCNA) [28] and tumor-metastasis associated proteins, Matrix metalloproteinase-7 (MMP-7) [29] were detected by Western blotting. The results showed that the expressions of PCNA and MMP7 were increased by EGF treatment (100 ng/mL, 24 h). Applying CHAG (500 or 1000 μg/mL) with EGF at the same time efficiently inhibited EGF-induced expressions of MMP-7 and PCNA (Fig. 3c–f). These results indicated that CHAG could inhibit the expression of migration and proliferation related proteins.

## Discussion

OC is one of the major causes of gynecologic cancer-related death, with an overall five-year survival rate of ~ 45% and an overall 10-year survival rate of 35% in the USA [30]. Currently, the mainstay of OC treatment includes cytoreductive surgery and platinum-based chemotherapy [30]. It is important to perform complete surgery for OC patients, and platinum resistance is the crucial problem in the treatment of these patients. In addition, postoperative tumor metastasis and recurrence cannot be ignored. There are four ways for OC cells to metastasize: direct spreading to the adjacent parts, lymph node metastasizing, blood metastasizing, and planting to other location. During the operation of tumor resection, it is easy to cause tumor cells to fall off and plant into the abdominal cavity or pelvic cavity. In addition to the above problems, adhesion is the most important complication of abdominopelvic surgery, causing some short- and long-term problems,** such as** infertility, small bowel obstruction and chronic pelvic pain [31]. The main strategies for adhesion prevention in gynecological surgery are focused on improvement of surgical technique and use of anti-adhesive agents, which fall into two major categories: pharmacological agents and barriers [32], and HA is one of them. HA is a naturally component of many body tissues and fluids, where it provides physically supportive and mechanically protective roles [33]. Various combinations of HA have been used for adhesion prevention. However, there were still some unsatisfactory results owing to rapid degradation of native HA. So, modification might be one of the effective ways to prolong the half-life of HA, such as crosslinking modification. In fact, CHAG applied in this paper had been reported to significantly reduce adhesion in abdominopelvic cavity after gynecological laparoscopic surgeries [12, 34]. However, since there was no data elucidating the effect of CHAG on the cells of gynecologic tumor, it is still unclear whether CHAG is safe enough for preventing postoperative adhesion in pelvic surgery of this kind of tumor.

In the in vitro study of this paper, we found that CHAG could inhibit the migration and invasion activities of ovarian cancer cells. And in the in vivo study of this paper, CHAG was administrated 2 h after inoculating ovarian cancer cells into pelvic cavity, which simulated the period that the inoculated cancer cells had been implanted and begun to proliferate in peritoneal cavity. After the first administration, CHAG was administrated weekly until the 4th week, which could simulate the period of mid-term growth of the implantation tumors. The exciting results was that the growth of the transplantation tumors was also efficiently inhibited, indicating that CHAG might be safe to be applied for preventing postoperative adhesion in surgical resection of OC.

In clinical, the effects of HA on tumor are different according to diverse molecular weights. HA synthase (HAS) generates predominantly high molecular weight HA (HMW-HA) with molecular weight between 200 and 2000 kDa, while the degradation generates different-sized HA polymers (or fragments), such as low molecular weight HA (LMW-HA; < 200 kDa) and HA oligomers [9]. In general, LMW-HA has pro-cancerous effect [35], whereas HMW-HA controls normal homeostasis and displays anti-cancerous activity [34, 35]. Furthermore, there are many controversial findings, which are related to the lack of consensus on size definition, the polydispersity of HA commercial products, and the use of HA from different animal or different tissues. And all the notices should be taken into account whenever a new study on HA is undertaken. The research results of this paper showed that crosslinked hyaluronan, as a HA polymer with boundless molecular weight, had an anti-tumor effects, which was in line with most previous data.

Next, it is worthy to investigate how does CHAG affect proliferation and metastasis activity of cancer cells. Owing to its physical adhesive characteristic, CHAG is speculated to cover the cells and block the interaction between ligands and their receptors. So, the effect of CHAG on the activation of the key growth factor receptor EGFR and its associated signaling molecules were detected. The result indicated that CHAG effectively blocked the EGF-induced phosphorylation/activation of EGFR, inhibited the EGF/EGFR-initiated activation of ERK, Akt and Rac1, and decreased the EGF-induced expression of PCNA and MMP7, suggesting that CHAG inhibited the growth and metastasis of OC cells via blocking the activation of EGFR and subsequent signaling. Our results are similar to the previous research that EGFR is reported to overexpressed in most ovarian cancer [36], and the activation of the EGFR pathway has impact on invasion and metastasis as well as cell survival through the MAPK/ERK, PI3K/AKT and **Rac1** pathways [37–41].

## Conclusion

In conclusion, our results demonstrated that CHAG could suppress the development of OC though blocking the activation of EGF-induced activation of EGFR and its downstream signal transduction. This provides evidence for safe application of CHAG in preventing postoperative adhesion of surgical resection of OC.

## Additional file

Additional file 1: **Figure S1**. The expression of EGFR in A2780 and SKVO3 cells. The celluar lysates were subjected to Western blotting with antibody against EGFR. Expression of β-actin was used at the same time as loading control. (TIFF 676 kb)

## Abbreviations

CHAG: Cross-linked hyaluronan gel
DMEM: Dulbecco’s modified Eagle’s medium
EGFR: Epidermal Growth Factor Receptor
GST-PBD: GST-Pak1 protein binding domain
HA: Hyaluronic Acid
HAS: HA synthase
HMW-HA: High molecular weight HA
IUA: Intrauterine adhesion
LMW-HA: Low molecular weight HA
MMP-7: Matrix metalloproteinase-7
OC: Ovarian carcinoma
PCNA: Proliferating cell nuclear antigen
SD: Standard deviation
SPF: Specific Pathogen Free
