# Supplementary figures and images for: Cross-linked hyaluronan gel inhibits the growth and metastasis of ovarian carcinoma
Source: J Ovarian Res. 2018 Mar 6;11:22. doi: 10.1186/s13048-018-0394-z (PMC5840805; doi:10.1186/s13048-018-0394-z)

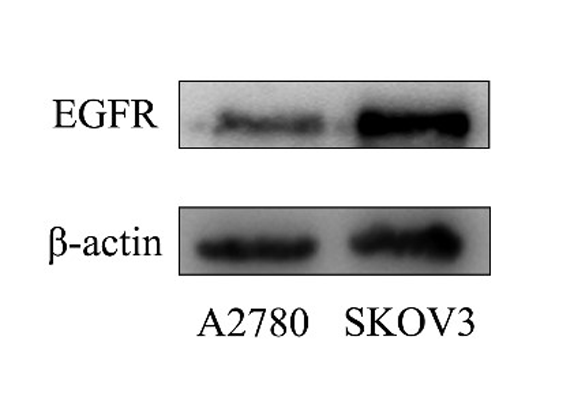

Supplement: Supplementary file 1 — Figure S1. The expression of EGFR in A2780 and SKVO3 cells. The celluar lysates were subjected to Western blotting with antibody against EGFR. Expression of β-actin was used at the same time as loading control. (TIFF 676 kb) [file 13048_2018_394_MOESM1_ESM.tif]
